# Relationship between team ranking and physical fitness in elite male handball players in different playing positions

**DOI:** 10.1038/s41598-024-53435-z

**Published:** 2024-02-08

**Authors:** Xiaobin Wei, Ji Zhang, Jian Wu, Chong Chen, Paweł Chmura, Peter Krustrup

**Affiliations:** 1 School of Strength and Conditioning Training, Beijing Sport University, Beijing, China; 2 China Institute of Sport Science, Beijing, China; 3 Beijing Muxiyuan Sports Technology School, Beijing, China; 4 Beijing Research Institute of Sports Science, Beijing, China; 5 Department of Team Games, Wroclaw University of Health and Sport Sciences, Wrocław, Poland; 6 Department of Sports Science and Clinical Biomechanics, SDU Sport and Health Sciences Cluster, University of Southern Denmark, Odense, Denmark; 7 Danish Institute for Advanced Study (DIAS), University of Southern Denmark, Odense, Denmark; 8 Sport and Health Sciences, College of Life and Environmental Sciences, University of Exeter, Exeter, UK

**Keywords:** Muscle, Occupational health, Human behaviour

## Abstract

The aim of this study was to identify the key physical indicators that affect game performance of male team handball athletes in national team handball tournaments and explore them further in relation to playing positions. A total of 150 male handball athletes were tested before a national tournament, using squat, bench press, 30-m sprint, vertical jump, pull-ups and abdominal endurance testing. Correlation analysis was used to examine the potential relationship between fitness level and tournament ranking. The results revealed significant differences in fitness variables among different playing positions (p < 0.05), excepted abdominal endurance and vertical jump (p > 0.05). 1RM squad performance was associated with team rankings (r = 0.289, p < 0.05). For individual playing positions, bench press of backs correlate with rankings (r = 0.354, p < 0.05). For goalkeepers, ranking was significantly positively correlated with 30-m sprinting (r = 0.604, p < 0.05). No other correlations were found (p > 0.05). To conclude, fitness level is to some extent related to team ranking with higher ranked teams having better scores in some of the fitness tests for all playing positions. The key predictors of performance in sport vary from position to position and practitioners need to differentiate between them to organize strength and conditioning training more precisely. More specialized fitness tests are also needed to assess athletes' competition ability.

## Introduction

Handball is a highly competitive sport that demands a combination of physical attributes such as speed, agility, strength, coordination and endurance^1,2^. The physical fitness of players is considered a key factor in determining the success of a team^3^, as it has been shown to be a significant predictor of team performance in elite team handball^4^. For example, throwing performance is considered to be a key factor in performance^5^, with expert athletes significantly better than novice athletes during throwing test^6^. In fact, the multifaceted nature of team handball necessitates a holistic approach to training and preparation. The intense nature of the sport, which involves rapid transitions between offense and defense, sudden changes in direction, and close-quarters physical contact, places unique physical demands on team handball players^7^. Thus, physical performance in elite team handball it not related to a single physical attribute; rather, it is the synergy of multiple attributes that creates a successful player and team.

Physical fitness has been found to be a significant predictor of team level in elite handball. For example, one study found that mean power output and jump performance were better in higher league ranking males handball team^8^. Pereira et al.^9^ also found that superior explosive power in higher level male handball players whereas no clear differences were observed for sprinting speed and agility. In contrast, Bruno et al.^10^ found that speed and agility was also better for higher level compared to lower level male team handball players. Regarding female team handball players, top elite athletes were found to be superior to sub-level athletes in fitness such as speed, explosive power and endurance^11^.

In conclusion, physical fitness seems to be a critical factor in determining the success of elite handball teams, and there is a positive relationship between several physical fitness variables and team ranking. However, the physical and physiology profile of handball players differ from position to position^12,13^, and the aforementioned studies did not distinguish between playing positions. Additionally, understanding the specific physical demands of different positions within team handball team is essential for developing targeted training programs. Each position requires a unique combination of skills and attributes to excel on the court. For instance, goalkeepers need explosive reflexes and exceptional hand–eye coordination to block shots, while wingers rely on speed and agility to outmaneuver their opponents on fast breaks.

Therefore, the present study collected fitness data of the athletes from all teams participating in the tournament and analysed them in relation to playing position. This will provide practitioners with a more detailed reference for strength and conditioning training and can bridge the gap in current research lacking in basic physical fitness studies affecting the game of handball.

Based on the highly physiological exertion characteristics of the handball and the results of the time-motion analysis in the studies, we hypothesized that (1) fitness level at the start of the season with positively correlate with team ranking in the tournament (2) key indicators of fitness vary between playing positions due to different tactical roles and tasks.

## Methods

### Sample and variable

A total of 150 elite male handball players took part in the study, and all players are national level. Positions were divided into goal keepers, back, pivot and line players, and all anthropometrical variables are presenting in Table 1. Athletes were tested with a test battery that including 6 fitness tests, i.e. squat, bench press, abdominal bridge, pull-ups, jump height and 30-m sprinting. The teams' tournament rankings are used for analysis. Subjects were informed about the risks and benefits of the fitness testing and the written informed consent was obtained from all subjects. The experiment conforms to the Declaration of Helsinki and the experimental protocols were approved by the ethics committee of China Institute of Sport Science.

**Table 1:** Basic information of participants.

Position	Age (yrs)	Height (cm)	Weight (kg)	BMI
All (n = 150)	23.9 ± 4.3	190.0 ± 5.5	86.8 ± 9.6	24.0 ± 2.2
Goal keepers (n = 19)	24.1 ± 4.2	193.2 ± 3.4	89.2 ± 8.2	23.9 ± 2.1
Back (n = 67)	24.4 ± 4.8	190.9 ± 5.6	87.7 ± 9.0	24.0 ± 2.1
Pivot (n = 24)	23.5 ± 3.9	190.9 ± 5.0	94.5 ± 9.0	25.9 ± 2.1
Line (n = 40)	23.4 ± 3.8	186.3 ± 4.6	79.4 ± 6.5	22.9 ± 1.8

### Procedure

**Figure 1:**
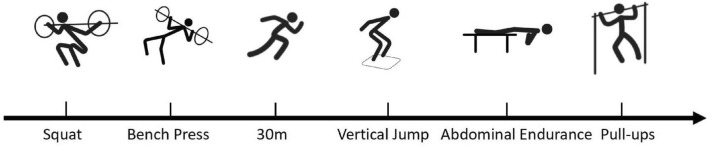
Fitness test procedure of players.

The fitness tests were conducted 3 days before the start of the tournament and was organised by the official handball association. All players had been through the test several times before and were familiar with the testing procedures. The test procedures started at 1 pm. Before all tests, a 10-min warm-up was conducted under the guidance of a professional fitness coach, and the warm-up included jogging and dynamic stretching. Besides, a re-warm-up was conducted before each test with special motions. The sequence of tests is as follows: squat, bench press, 30-m sprint test, vertical jump, abdominal endurance, pull-ups (Fig. 1). To ensure reliable data collection, each test was measured by the same testers with extensive testing experience. We also used a systematic approach for data recording, with another person double-checking the recorded test results.

#### Squat

This test involves a deep squat rack, barbell bar, elastic rope and a set of 200 kg/set of barbell piece. Before the test, athletes measure their weight and record to one decimal point. During the warm-up, athletes perform 6–12 reps at 50–75% maximum intensity and 1–3 reps at 75–90% maximum intensity, completing a total of about 5–6 sets. When the athlete's warm-up is complete, the formal test is conducted with the following criteria: the subject stands with both feet slightly wider than shoulder width, and the toes can be externally rotated 15°–30°. Squat until the front side of the thigh reaches or is below the horizontal line and then stand up with force. Each athlete has 2 trials and the best performance was recorded as the rest results, measured in kilogram.

#### Bench press

This test involves a bench press rack, barbell bar, elastic rope and a set of 160 kg/set of barbell piece. When the athlete has completed the warm-up, the formal test is conducted with the following criteria: the test athlete lies supine in the bench press rack, adjusts to the proper height, feet on the ground, spine against the bench press chair, head flat on the bench press chair, occipital bone touching the chair surface, hands gripping the barbell bar at the proper width. Pick up the barbell, bend the arms downward, elbows should be at or less than 90°, then pull-ups ward until both elbows are fully extended. After a full warm-up, each athlete has two trials and the best performance was recorded as the test result.

#### 30-m sprint

The test was performed according to the following standards: standing start, the starting point and the first light gate distance to be less than one meter, with the fastest speed to complete the 30-m sprinting distance. Each athlete will have two trials, and the best performance was recorded as the test results. The 30-m sprint performance was measured in seconds and reported with 2 decimals. Athletes stretch before the test, focusing on stretching the muscles around the hip joint, including the gluteus maximus, hamstrings, calf triceps and trunk muscles, while then warming up with a sharp stop and start action pattern, with a 10-min warm-up. The test was conducted by photoelectric cells (Brower timing system, USA).

#### Vertical jump

An 8-min warm-up was conducted prior to the test: Athletes stretch the lower limb hip, knee and ankle joints before the test and perform the corresponding movement pattern training as a warm up, which lasts 8 min. The official test is conducted after the warm-up activity and the criteria are as follows: the athlete stands with both feet on the vertical jumping mat, swings the arms in place and jumps upwards as far as possible and lands back in the original position, keeping the body straight during the vacating process. 2–3 attempts were allowed before the test, and each athlete has two trials and the best performance was recorded as the test result. The test equipment is a vertical jump mat (IRONMAN, China).

#### Abdominal endurance

The subject lies in the prone position on the bench with the torso suspended, the anterior superior iliac spine on the edge of the bench, the arms crossed over the chest and the lower legs held in place with the help of another person, keeping the body in a horizontal position, not more than 15° above or below the horizontal. A warning was given on the first occurrence and the test was stopped on the second occurrence if the following occurs. For this test, only one trial was given.

#### Pull-ups

This test involves a bar, high bench and non-slip powder. Before the test, the athlete stretches the upper limb muscles, moves the shoulder and elbow joints, and becomes familiar with the bar. After an 8-min warm-up, the test is conducted using the following standards: The subject will hold both hands squarely, spaced slightly wider than shoulder width, pull up to the jaw and cheek line over the bar, and the elbow joint must be straight when lowering. Additional movement assistance is allowed. The number of times the athlete completes the movement as required was noted as the test result. Pull-ups with obvious technical quality problems were not counted.

### Statistical analysis

Data were expressed as mean ± standard deviation. Statistical processing using SPSS 26.0(IBM, Chicago, USA). Normality was performed by Kolmogorov–Smirnov tests. One-way ANOVA with LSD post-hoc test was used for analyzing the fitness difference between various position. Applicable partial eta squared (η_p_^2^) reflects the effect amount: 0.04, 0.25 and 0.64 are the critical values of small, medium and large effect amounts, respectively. Spearman correlation coefficients (r) were used to determine association between tests and ranking. The criteria for correlation are as follows: r = 0.10–0.29 (small), 0.30–0.49 (medium), 0.50–0.69 (large), 0.70–0.89 (very large), 0.90–0.99 (almost perfect), and 1.00 (perfect)^14^. Significant level was set as p < 0.05. Moreover, to reduce the false discovery rate, the alpha level was corrected by using the Benjamini–Hochberg procedure (FDR < 5%).

## Results

Table 2 shows the fitness test results for the various playing position. ANOVA tests revealed significant differences were found in the performance of the fitness tests among the playing positions (p < 0.05, η_p_^2^ = 0.054–0.172), excepted abdominal endurance and vertical jump (p > 0.05, η_p_^2^ = 0.005–0.045).

**Table 2:** Fitness test results in different playing positions.

Fitness	Position				F	p	η_p_^2^
	Goal keeper	Back	Pivot	Line			
Squat	136.5 ± 22.8^b^	138.7 ± 21.5^d^	152.1 ± 20.7^bdf^	134.7 ± 21.0^f^	3.589	0.015	0.069
Bench press	102.7 ± 14.3^b^	103.8 ± 16.3^d^	108.3 ± 15.5^bdf^	92.8 ± 14.3^f^	6.303	0.000	0.115
Abdominal endurance	117 ± 14.0	116.1 ± 28.8	112.1 ± 12.5	115.1 ± 15.8	0.237	0.870	0.005
Vertical jump	70.5 ± 5.7	72.9 ± 7.6	69.6 ± 6.6	73.7 ± 6.9	2.288	0.081	0.045
30-m	4.26 ± 0.11^ac^	4.14 ± 0.13^ae^	4.23 ± 0.12^ef^	4.11 ± 0.14^cf^	10.090	0.000	0.172
Pull-ups	23.1 ± 4.3	22.0 ± 6.0^e^	21.0 ± 4.2^f^	24.3 ± 3.6^ef^	2.759	0.044	0.054

^a^Denotes significant difference with goal keeper and back.

^b^Denotes significant difference with goal keeper and pivot.

^c^Denotes significant difference with goal keeper and line.

^d^Denotes significant difference with back and pivot.

^e^Denotes significant difference with back and line.

^f^Denotes significant difference with pivot and line.

**Figure 2:**
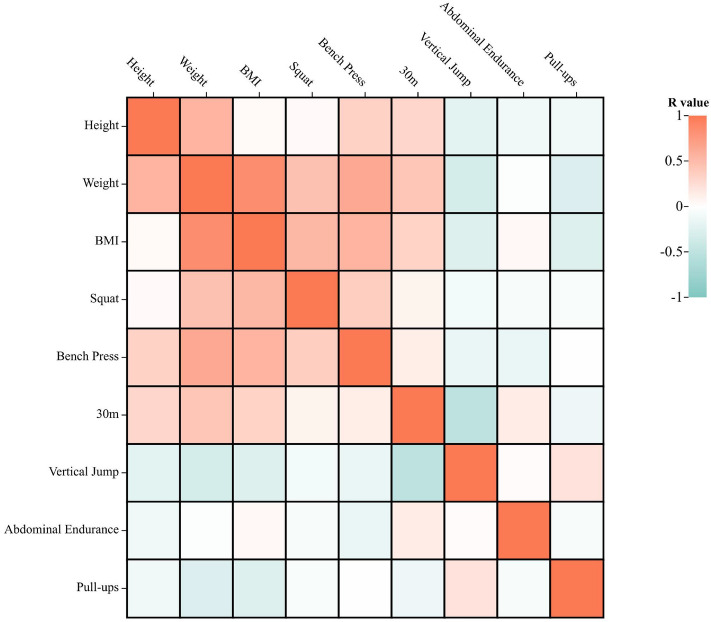
Correlation thermograms for athletes' physiological characteristics.

Figure 2 shows a thermogram of the correlations of physiological characteristics of the team handball athletes. For anthropometrics, height was positively correlated with body weight bench press (r = 0.30–0.56, p < 0.05) and negatively correlated with vertical jump height (r = − 0.22, p < 0.05). Body weight was correlated with all data except abdominal endurance, with a negative correlations with vertical jump height (r = − 0.34, p < 0.05) and number of pull-ups (r = − 0.28, p < 0.05), and positive correlations with the other data (r = 0.43–0.85, p < 0.05). BMI was significantly correlated with all data except abdominal endurance, where it was negatively correlated with vertical jump height (r = − 0.27, p < 0.05) and number of pull-ups (r = − 0.27, p < 0.05), and positively correlated with the other data (r = 0.33–0.85, p < 0.05). For physical fitness tests, squat performance was positively correlated with bench press (r = 0.37, p < 0.05), bench press was negatively correlated with vertical jump height (r = − 0.16, p < 0.05) and abdominal endurance (r = − 0.17, p < 0.05), 30-m sprint was negatively correlated with vertical jump height (r = − 0.52, p < 0.05), and vertical jump height was positively correlated with number pull-ups (r = 0.21, p < 0.05).

Table 3 presents the correlation analysis between the fitness test and the competition ranking. Bench press performance was significantly correlated with ranking (r = 0.29, p < 0.05), while the other tests were not correlated with ranking (p > 0.05).

**Table 3:** Correlations between fitness and ranking.

	r	P (adjusted)
Squat	0.289	0.000*
Bench press	− 0.046	0.718
Vertical jump	− 0.140	0.176
Abdominal endurance	− 0.020	0.807
30-m	− 0.043	0.718
Pull-ups	− 0.143	0.176

*Denotes p < 0.05.

In terms of correlating test scores with rankings at different positions, the results from Table 4 show that bench press of back correlate with ranking (r = 0.35, p < 0.05). For goalkeepers, the ranking was positively correlated with 30-m sprint performance (r = 0.60, p < 0.05). No correlations were found for the other data(p > 0.05).

**Table 4:** Spearman correlation between team ranking and players’ fitness in different position.

Fitness	Position			
	Goal keeper	Back	Pivot	Line
Squat	0.183	0.354*	0.194	0.393
Bench press	0.072	0.013	− 0.093	− 0.268
Vertical jump	− 0.518	− 0.033	− 0.451	0.066
Abdominal endurance	0.379	− 0.183	0.002	0.031
30-m sprint	0.604*	− 0.115	0.075	− 0.293
Pull-ups	− 0.320	− 0.080	− 0.150	− 0.034

*Denotes p < 0.05 (adjusted).

## Discussion

The present study investigated the importance of selected fitness variables on the performance of handball players by correlating their pre-tournament fitness test results with their ranking in a national tournament. We found some differences in fitness test results between positions, and fitness indicators were related to ranking for all playing positions, with fitness level being better for higher ranked teams, partly consistent with our hypothesis.

Our results show difference among different position in terms of fitness, in consistence with most previous studies^1,15,16^ but contrast to the study of Chaouachi et al.^1,15–17^. However, the effect size results show that the degree of fitness differences in position is not particularly high. The reasons for the inconsistent findings may lie in the differences in the number of subjects selected, as well as in the choice of specific test content. On the other hand, the anthropometric differences in handball players were minimal^18^, and the match physical profile differences were smaller than in other team sports^19^. In the study of Nikolaidis et al.^20^, there was no difference in most fitness between positions for adult athletes. In fact, the correlation between anthropometrics and fitness which shows in Fig. 2, and in particular strength, is high, in line with previous studies^21,22^. The small anthropometrical differences between athletes at different positions in this study may also account for the small differences in positional fitness.

In addition, we found that only the squat 1RM was associated with team ranking. However, the correlation coefficient (r = 0.289) indicates that the higher the 1RM squat, the lower the ranking of the team. The reason for this may be that explosive power is extremely important for handball players^3,23^, and too much maximum strength can negative affect explosive power according to force–velocity relationship^24^. This was also reflected in the correlation analysis of the fitness tests (Fig. 2), which showed a negative correlation between bench press and vertical jump height. In a previous study it was shown that the 1RM squat of national level athletes was lower than the Division 1 athletes^25^. This finding suggests that excessive maximum strength in elite handball players may negatively affect the player's match performance and therefore practitioners need to balance maximum strength with explosive power.

Regarding to different position, the backs and the lines are in line with the overall trend. Whereas only goalkeeper's 30-m sprint showed a moderate correlation with ranking. Interestingly, while the raw p-values showed a significant correlation between standing jumps and rankings for goalies and wingers, the adjusted p-values did not reach the significance level. The height of the vertical jump is one of the indicators of explosive power^26^. The high explosive power of goalkeepers and pivots means that they are able to make quick, split-second tackles and throws^27^, which are important for achieving an advantage in the game^28^. The reason that no significant correlation was found between vertical jump height and ranking after adjust P value may be related to the fact that vertical jump height is not the best predictor of explosive power^29^ and the differences in jump characteristics between test and games^30^. Furthermore, only the goalkeeper's 30-m sprint performance was correlated with ranking, is consistent with the many previous studies that have found that speed ability is not a determinant of handball player level^9,25^. Although some studies have found a correlation between speed ability and athletic level in handball players, the sprint distances used in that study were much shorter^31^. In fact, handball players do not sprint as often and have shorter sprint distances^3,19^, thus speed ability may not be a key indicator to differentiate an athlete's level. The above results suggest that practitioners need to individualize players` physical training according to the characteristics and positions in the game.

Finally, 1RM bench press, pull-ups and abdominal endurance tests do not correlate with ranking, indicating these capacities are not among the most vital indicators of athlete's capacities. The r value of the indicator that is significantly correlated with the ranking is not very high also suggests that other physical abilities (e.g. aerobic capacity, agility), technical and tactical ability may be more important for handball players^32,33^. Therefore, coaches should identify the specific needs of their handball players and organise their training in a way that is appropriate to their level.

The findings of this study provide valuable practical implications for coaches, sports scientists, and practitioners involved in the fitness training of elite handball players. Firstly, understanding the correlations between fitness variables and performance can guide more informed decision-making in the design of training programs. For example, training that focuses too much on improving maximal strength may affect an athlete's explosiveness and impair match performance. Secondly, coaches should design customized strength and conditioning programs for handball athletes based on their playing positions. Adopt an individualized approach to training, taking into account the unique requirements of each position for optimal performance.

One of the limitations of this study is that the fitness tests lack of agility and aerobic capacity contents. In addition, there is a possibility that athletes may not try their best during the test due to the vulnerability of the 1RM test and the short interval between the test and the competition. The lack of specialization in the content of the test may affect the conclusions of the study. Future research could extend the test`s scope and specialization, and conduct studies with female athletes to discover more specific predictors of match performance. Moreover, we lack of time-motion results during games, which is crucial for performance analysis in team sport^34^. Future research could incorporate metrics of motion analysis to provide a deeper insight for practitioners.

## Conclusion

The present data showed that fitness level is to some extent related to team ranking with higher ranked teams having better scores in some of the fitness tests for all playing positions. However, the low level of correlation suggests that physical fitness has a limited impact on match performance, and that other factors such as technique and tactics may have more of an impact on match performance. The findings of this study have practical implications for coaches and strength and conditioning specialists working with handball athletes.

It is important to note that the study found the fitness indicators that significantly correlate with ranking are different for athletes in different playing positions, indicating that a one-size-fits-all approach to strength and conditioning may not be effective for improving game performance in Team Handball. Coaches should instead focus on developing training programs that are target to the specific needs of each athlete based on their position and individual strengths and weaknesses.

## Data Availability

Data can be requested from the corresponding author on reasonable request.
